# A randomized trial to assess retention rates using mobile phone reminders versus physical contact tracing in a potential HIV vaccine efficacy population of fishing communities around Lake Victoria, Uganda

**DOI:** 10.1186/s12879-018-3475-0

**Published:** 2018-11-21

**Authors:** Noah Kiwanuka, Juliet Mpendo, Stephen Asiimwe, Julius Ssempiira, Annet Nalutaaya, Betty Nambuusi, Mathias Wambuzi, Brian Kabuubi, Annemarie Namuniina, Frederick Oporia, Annet Nanvubya, Ali Ssetaala

**Affiliations:** 1 School of Public Health, College of Health Sciences, Makerere University, P.O.Box 7072, Kampala, Uganda; 2 Clinical Trials Unit, College of Health Sciences, Makerere University, Kampala, Uganda; 3 UVRI-IAVI HIV Vaccine Program, Entebbe, Uganda; 4 Kabwohe Clinical Research Center (KCRC), Kabwohe, Uganda

**Keywords:** Fishing communities, Retention (follow-up) rates, HIV vaccine

## Abstract

**Background:**

High retention (follow-up) rates improve the validity and statistical power of outcomes in longitudinal studies and the effectiveness of programs with prolonged administration of interventions. We assessed participant retention in a potential HIV vaccine trials population of fishing communities along Lake Victoria, Uganda.

**Methods:**

In a community-based individual randomized trial, 662 participants aged 15–49 years were randomized to either mobile phone or physical contact tracing reminders and followed up at months 1, 2, 3, 6, 12 and 18 post-enrolment. The visit schedules aimed at mimicking a vaccine efficacy trial representing an early interval (months 1–6) where most vaccinations would be administered and a later period of post-vaccination follow-up. The primary outcome was retention measured as the proportion of post-baseline follow up visits completed by a participant. Retention was estimated in early and later follow-up intervals, and overall for all the six follow-up visits. Adjusted differences in retention between the study arms were determined by multivariable logistic regression using Stata® 14. One participant was later dropped from the analysis because of age ineligibility discovered after enrolment.

**Results:**

Of the expected total follow up visits of 3966 among 661 participants, 84.1% (3334) were attained; 82.1% (1626/1980) in the phone arm and 86% (1708/1986) in the physical tracing arm (*p* = 0.001). No statistically significant differences in retention were observed between the study arms in the first 6 months but thereafter, retention was significantly higher for physical contact reminders than mobile phones; 91.5% versus 82.1% (*p* < 0.0001) at month 12 and 82.8% versus 75.4%, (*p* = 0.021) at month 18. Controlling for sex, age, education, occupation, community location, length of stay and marital status, the odds of good retention (completing 5 out of 6 follow-up visits) were 1.56 (95% CI;1.08–2.26, *p* = 0.018) for physical contact tracing compared to mobile phone tracing. Other statistically significant predictors of good retention were residing on islands and having stayed in the fishing communities for 5 or more years.

**Conclusions:**

Among fishing communities of Lake Victoria, Uganda, 84% of follow-up visits can be attained and participant retention is higher using physical contact reminders than mobile phones.

**Trial registration number:**

PACTR201311000696101 (http://www.pactr.org/). retrospectively registered on 05 November, 2013.

## Background

HIV still remains a significant problem especially in sub-Saharan Africa where the rate of new HIV infections is still unacceptably high despite the increased availability of proven interventions and attainment a tipping point (a condition where the number of persons initiating anti-retroviral therapy is greater than the number of new infections). An effective HIV preventive vaccine is urgently needed to complement the current HIV prevention and treatment strategies. A mathematical modeling study has showed that the UNAIDS 95–95–95 target alone could avert about 25.3 million new HIV infections and the addition of a vaccine with 50% efficacy and 70% coverage to the 95–95–95 target could prevent 31 million infections by 2035. Moreover, an HIV vaccine alone was predicted to prevent 17 million incident HIV infections beyond status quo interventions [1]. A number of vaccine candidates are currently under development and have either reached human trials stage or will do so soon [2, 3].

Trials evaluating candidate vaccines require high levels of adherence to scheduled study visits and investigational products. Levels of participant retention (follow-up rates) affect the validity of study outcomes, the statistical power to detect meaningful differences, and the efficacy and effectiveness of intervention [4, 5]. Attainment of high retention rates requires minimizing loss to follow-up (LTFU) rates and this is a challenge to research studies and implementation programs. Barriers to retention include transport, inflexible work schedules and study visits, forgetting research appointments, participant/patient fatigue, demanding domestic responsibilities, mistrust of researchers and uncertainty of study outcomes [6–8]. Observational studies among potential populations for HIV vaccine efficacy trials have reported retention rates of 69–85.2% at 12 months and 76% at 18 months of follow-up [9–14]. These rates are below the desired minimum of 90%. A randomized trial in Kenya that assessed the effect of mobile phone use (text messages/audio calls) and in-person (physical contact) reminders on visit attendance did not have a separate physical contact arm making it impossible to determine the effect of the latter [15]. Other limitations of the aforementioned studies include the absence of follow-up schedules that mimic vaccine efficacy trials and the hypothetical assessment in the absence of an actual HIV vaccine efficacy trial. To address some of the above limitations, we conducted a randomized trial of mobile phone reminders versus physical contact reminders, to assess retention rates using a follow-up schedule that mimicked an HIV vaccine efficacy trial.

## Methods

### Study design and population

Between April 2012 and April 2014, we conducted a community-based individual randomized unblinded two arm trial in four fishing communities (2 islands and 2 mainland lakeshore villages) along Lake Victoria, Uganda, and assessed retention rates using mobile phone (short-text message and or audio) reminders versus physical contact tracing. The objective of the trial was to determine whether retention rates differed by method of reminding - mobile telephones (calls and text messages) versus physical contact tracing. The primary outcome was the proportion of post-baseline follow up visits completed by each participant. A follow up visit was counted as completed if a participant seen and interviewed by the study team on a pre-scheduled date or within the visit window period. Six hundred and sixty two participants aged 15–49 years were enrolled and randomized to either mobile phone or physical tracing. Participants in both arms had a total of six post-enrolment follow up visits at months 1, 2, 3, 6, 12 and 18. Each scheduled visit had a window of ± one week from the scheduled date. The visit schedule was designed to mimic an actual HIV vaccine efficacy trial follow up schedule, representing two periods typically encountered in actual HIV vaccine trials; the first six months where vaccinations are administered (earlier interval) and the post 6 months period for assessment of endpoints/outcomes (later interval). Participants in both arms were reminded twice about an upcoming visit; 7 and 3 days to the scheduled date. In the physical tracing arm reminders were done community health mobilizers (CHMs) who were members of local village health teams via face-to-face discussions. Approximately two weeks prior to each visit, CHMs were given lists of participants in their villages with dates of scheduled visits and their associated window intervals. CHMs were instructed to thank participants for their previous visits participation and to remind them of the upcoming next visit including the day and date. Phone call reminders were done by a member of the research team who was stationed at the central coordination office and had no direct physical contact with participants. Each participant in the phone arm was called and informed about an impending follow up visit. If a participant’s audio call did not go through, further attempts were made twice a day (one in the morning and the other in the afternoon) till the expiration of the visit window interval. Short text messages were sent to all phone arm participants at the lower end of the window interval and one day prior to the scheduled date. The text message was *“Hello, thank you for participating in the STAR research study. We are reminding you of your next study visit which is on (day and date). STAR study team.”* All messages were in Luganda, the most commonly spoken local language in the study communities.

### Sample size determination

Our sample size estimation was based on a previous study in the similar communities that found a cumulative retention of 80% at 18 months of follow up. We hypothesized a retention of 80% (or *p* = 0.8) among the physical contact arm and an increase of 8.75% mobile phone arm 18 months post-baseline. Using the sample size formula for binary outcomes, a two-sided alpha of 0.05, power of 80%, and adjusting for a non-response proportion of 15% and cross-over – a drop in rate (R_I_) of 5% and dropout rate (R_O_) of 3%, we estimated a total sample size of 662 participants (331 per arm).

### Screening and eligibility criteria

*Inclusion criteria*: Eligible participants had to be: 1) aged 15–49 years; 2) capable of providing written informed consent/assent; 3) HIV sero-negative; 4) at risk of HIV infection defined as having had any of the following in the past 3 months: a symptom of a sexually transmitted infection (STI), more than one sexual partner, a new sexual partner, or sex with a worker; 5) willing to undergo HIV testing and receive HIV results; 6) owning a cell phone; and 7) willing to provide locator information. *Exclusion criteria*: Participation in another interventional or behavioral study at the time of enrolment and presence of any condition which, in the opinion of the investigator or designee, would interfere with achieving the objectives of the study.

### Randomization

Participants were randomized to either phone or physical contact reminder using permuted block randomization with blocks of size of 6 and a 1:1 allocation ratio. Randomization numbers were generated using Stata™ software version 11 and individual random assignment sheets were placed and sealed in opaque envelopes in batches of multiple blocks to minimize predictability that would occur if single blocks were used. Generation of randomization numbers and their concealment was done by a Biostatistician based at the Makerere University Clinical Trials Unit who never had contact with participants. She provided concealed envelopes to the Study Coordinator in numbers corresponding to the expected enrolments for given time periods. Accountability for earlier randomization envelopes was given prior to release of subsequent ones. Each consenting participant was asked by a field data collector to select an envelope which s/he opened to see the randomization number and study arm. All randomization numbers were monitored and logged daily in the field, and electronically via a data base at a data management center.

### Laboratory testing

HIV-1 serostatus was determined by rapid HIV tests performed in the community by certified laboratory technologists and EIA confirmation in the laboratory at Uganda Virus Research Institute. Blood samples were first tested with Determine® HIV assay (Alere Medical Co., Ltd., Chiba, Japan), and if negative, results were reported as negative. Determine® positive samples were then tested with HIV 1/2 Stat-Pak® assay (Chembio Diagnostic Systems, Inc. Medford, NY, USA), and if positive too, results were reported as positive. But if negative on Stat-Pak®, Uni-Gold™ HIV test (Trinity Biotech plc, Bray, Ireland) was used as a tie-breaker.

### Statistical analysis

Participants socio-demographic characteristics were summarized in frequencies and compared between those screened and enrolled, and across study arms using chi-square and Fisher Exact tests for categorical variables. Retention was estimated as the proportion of visits that a participant completed, divided by the total number of expected follow up visits. The follow-up period was categorized into the early interval (comprising the first 4 post-baseline visits of month 1, 2, 3 and 6) corresponding to the intensive phase of a vaccine trial, and a later period (the last 2 visits of month 12 and 18) which represented the post-vaccination follow-up phase. Retention was also estimated at each month of follow-up, by period interval (earlier and later), and overall for all the six follow-up visits. We defined good retention as completion of at least five of the six scheduled follow-up visits. We assessed if retention differed between study arms at each follow up visit, across socio-demographic and behavioral characteristics in early and later follow-up intervals, using intention-to-treat analysis. Crude and adjusted odds ratios of retention were estimated using multivariable logistic regression modeling, testing for differences between the study arms and associations with various socio-demographic and behavioural characteristics. Covariates were included in the multivariable models based on biological plausibility and a bivariate statistical significance at an alpha (α) of < 0.15. Models were built using the stepwise logical model building approach and the final model adjusted for age, gender, education, marital status, occupation, location of community and duration of stay in the communitiy. All statistical analyses were performed with Stata® 14 (StataCorp, College Station, TX) software. This article adheres to CONSORT guidelines.

### Ethics and consent

The protocol was reviewed and approved by the Uganda Virus Research Institute Research and Ethics Committee, and the Uganda National Council for Science and Technology. Written informed consent was obtained from all study participants aged at least 18 years. Participants aged less than 18 years (minors) gave assent in addition to parent or guardian consent. Persons who were found HIV sero-positive at screening or during the follow up visits were referred to ART clinics for further management. HIV treatment and care was provided by ART clinics in the study communities that were operated by the UVRI-IAVI HIV Vaccine Program and MildMay Uganda. Participants who opted for non-Program clinical services were referred to Entebbe General Hospital, a public health facility that serves the region where the study was done.

## Results

### Baseline characteristics of the study population

**Fig. 1:**
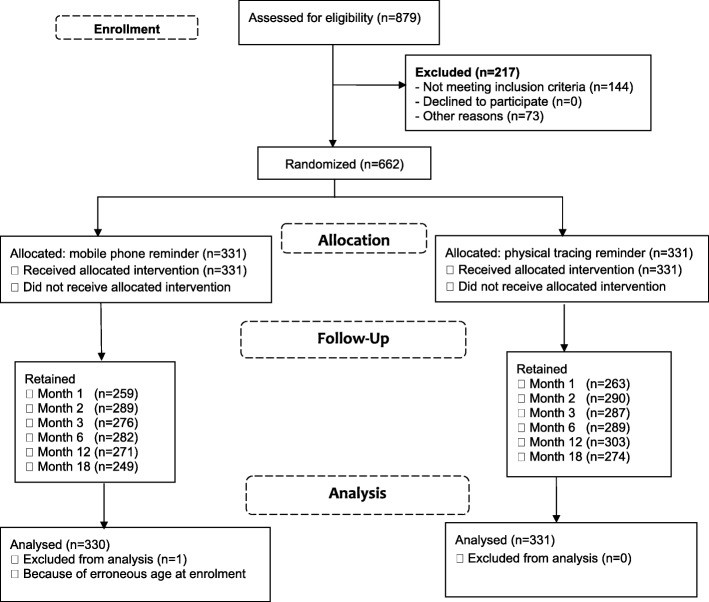
Trial profile

A total of 879 potential participants were screened of whom 83.6% (735) were found eligible and 662 were enrolled and randomized (Fig. 1). Among those excluded, 144 did not meet the inclusion criteria while 73 were eligible but the sample size had already been attained; only one screened participant did not have a mobile phone. During the trial, we discovered that one participant in the phone arm was wrongly enrolled by attaining his 15th birthday; he was dropped from the final analysis leaving a total of 661 participants. The major reason for study ineligibility was HIV seropositive at screening, 14.6% (128/879). There were no statistically significant differences between the enrolled and screened participants with regard to age, education, tribe, occupation, community of stay (island or mainland), and length of stay in the community (Table 1). However, statistically significant differences were observed with regard to gender, religion and marital status; more males, (*p* = 0.004), Muslims (*p* = 0.035) and marrieds (*p* = 0.024). Participants randomized in the two study arms did not differ in baseline socio-demographic characteristics except occupation; a higher proportion of housewives was enrolled in phone arm while more bar/lodge/restaurant workers were recruited in the physical contact tracing arm. Of the approximately 1324 audio calls made to the phone arm participants during the study, 12% (159) did not go through and reported phone loss at the end of the study was about 8.2%. This is likely an underestimate given that we captured this info from only those who called in to the study office using other numbers and reported phone loss.

**Table 1:** Baseline socio-demographic and behavioural characteristics of screened and enrolled participants

	Screened			Enrolled		
	Enrolled No. (%)	Not enrolled No. (%)	*P* value	Phone Arm No. (%)	Physical Arm	*P* value
All Participants	661 (75.2)	218 (24.8)		330 (49.9)	331 (50.1)	
Socio-demographics						
Sex						
Male	403 (78.7)	109 (21.3)		199 (49.4)	204 (50.6)	
Female	258 (70.3)	109 (29.7)	0.004	131 (50.8)	127 (49.2)	0.726
Age at enrolment (years)						
15–19	49 (75.4)	16 (24.6)		26 (53.1)	23 (46.9)	
20–24	175 (77.8)	50 (22.2)		88 (50.3)	87 (49.7)	
25–34	320 (74.8)	108 (25.2)		159 (49.7)	161 (50.3)	
35–49	117 (73.1)	43 (26.9)	0.749	57 (48.7)	60 (51.3)	0.964
Highest education level						
None	34 (69.4)	15 (30.6)		21 (61.8)	13 (38.2)	
Primary	379 (74.5)	130 (25.5)		187 (49.3)	192 (50.7)	
Secondary	230 (77.2)	68 (22.8)		117 (50.9)	113 (49.1)	
Post-secondary	18 (78.3)	5 (21.7)	0.619	5 (27.8)	13 (72.2)	0.134
Religion						
Catholic	256 (71.5)	102 (28.5)		120 (46.9)	136 (53.1)	
Protestant/Anglican	170 (74.6)	58 (25.4)		78 (45.9)	92 (54.1)	
Muslim	146 (82.9)	130 (17.1)		81 (55.5)	65 (44.5)	
Pentecostal/Adventist	89 (76.1)	28 (23.9)	0.035	51 (57.3)	38 (42.7)	0.122
Ethnicity/tribe						
Baganda	325 (75.4)	105 (24.6)		167 (51.4)	158 (48.6)	
Non-Baganda	336 (75.0)	112 (25.0)	0.938	163 (48.5)	173 (51.5)	0.460
Occupation						
Fishing/Fishing related^a^	270 (75.0)	90 (25.0)		139 (51.5)	131 (48.5)	
Shop/Market	46 (71.2)	18 (28.1)		19 (41.3)	27 (58.7)	
Bar/Lodge/Restaurant	70 (72.2)	27 (27.8)		25 (35.7)	45 (64.3)	
Farming	73 (78.5)	20 (21.5)		39 (53.4)	34 (46.6)	
Housewife	66 (82.5)	14 (17.5)		42 (63.6)	24 (36.4)	
Others^b^	136 (73.5)	49 (26.5)	0.553	66 (48.5)	70 (51.5)	0.026
Community location						
Mainland	353 (73.1)	130 (26.9)		179 (50.7)	174 (49.3)	
Island	308 (77.8)	88 (22.2)	0.109	151 (49.0)	157 (51.0)	0.666
Duration in community at enrolment (years)						
Less than 2	85 (72.7)	32 (27.3)		39 (45.9)	46 (54.1)	
2–4	259 (76.6)	79 (23.4)		129 (49.8)	130 (50.2)	
5–10	192 (74.1)	67 (25.9)		101 (52.6)	91 (47.4)	
More than 10	125 (75.8)	41 (24.2)	0.810	61 (48.8)	64 (51.2)	0.760
Marital status						
Not married	209 (69.7)	91 (30.3)		103 (49.3)	106 (50.7)	
Married monogamous	325 (78.1)	91 (21.9)		161 (49.5)	164 (50.5)	
Married polygamous	127 (77.9)	36 (22.1)	0.024	66 (52.0)	61 (48.0)	0.875

^a^Fishing, fishmongers, fish processing, boat maker/owner, ^b^Construction/Mechanic/Government/Clerical

### Retention

For each enrolled participant there were six scheduled post-baseline follow up visits, making a total of 3966 follow-up visits among the 661 enrolled participants. Overall, 84.1% (3334) follow-up visits were attained; 82.1% (1626/1980) in the phone arm and 86% (1708/1986) in the physical tracing arm (*p* = 0.001). Retention was 79% at month one, 87.6% at month two, 85.2% at month three, 86.4% at month six, 86.8% at month 12, and 79.1% at month 18.

**Fig. 2:**
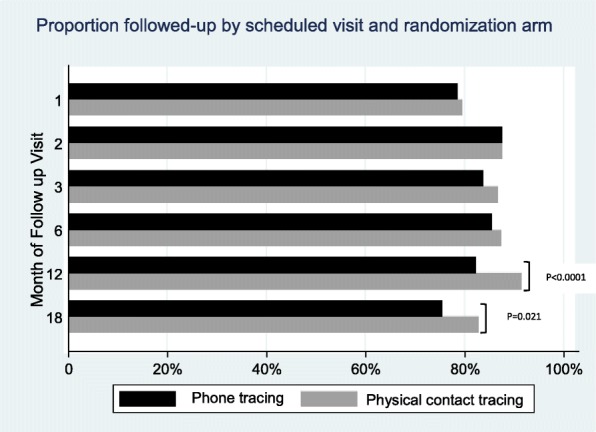
Proportion of follow-up at scheduled visits by study arm

Retention did not differ between study arms during the first 6 months of follow-up (Fig. 2). Thereafter, physical contact reminding had significantly higher retention than mobile phones at month 12 (91.5% versus 82.1%, *p* < 0.0001) and at month 18 (82.8% versus 75.4%, *p* = 0.021). In early follow-up phase, 64.3% (425) of participants completed all the four scheduled visits, and retention was significantly higher among participants from island communities compared to their counterparts on the mainland (72.4% versus 57.2%, *p* < 0.0001) and in participants who had stayed in fishing communities for more than 10 years (72.8%, *p* = 0.024) relative to those with less than 10 years of residence. During this phase of follow-up, no differences in retention rates were observed between the two study arms nor by other socio-demographic characteristics.

During the later follow-up period, 75.2% (497) completed the two scheduled visits. In that interval, retention was higher in the physical contact arm than in the mobile phone arm (80.1% versus 70.3% respectively, *p* = 0.004) and those in polygamous marriages (81.1%) relative to monogamous marriages (76.9%) and the unmarried (68.9), *p* = 0.026. Retention was 84.8% among participants who had stayed in fishing communities more than 10 or more years, 83.3% in those with 5–10 years, 70.3% in those with 2–4 years, and 57.7% among ones with less than two years of stay in the communities, *p* < 0.0001 (Table 2).

**Table 2:** Retention in the early and later phases by socio-demographics characteristics

Characteristics	Intensive Early Phase			Later Phase		
	4/4 *n* (%)	< 4/4 *n* (%)	*P* value	2/2 n (%)	< 2/2 n (%)	*P* value
All Participants	425 (64.3)	236 (35.7)		497 (75.2)	164 (24.8)	
Study Arm						
Phone tracing	215 (65.2)	115 (34.8)		232 (70.3)	98 (29.7)	
Physical contact tracing	210 (63.4)	121 (36.6)	0.647	265 (80.1)	66 (19.9)	0.004
Sex						
Female	171 (66.3)	87 (33.6)		198 (76.7)	60 (23.3)	
Male	254 (63.0)	149 (37.0)	0.395	299 (74.2)	104 (25.8)	0.459
Age group						
15–19	25 (51.0)	24 (49.0)		130 (74.3)	45 (25.7)	
20–24	121 (69.1)	54 (30.9)		35 (71.4)	14 (28.6)	
25–34	201 (62.8)	119 (27.2)		235 (73.4)	85 (26.6)	
35–49	78 (66.7)	39 (33.3)	0.105	97 (82.9)	20 (17.1)	0.194
Location of Community						
Mainland	202 (57.2)	151 (42.8)		274 (77.6)	79 (22.1)	
Island	223 (72.4)	85 (27.6)	< 0.0001	223 (72.4)	85 (27.6)	0.121
Occupation						
Fishing/Fishing related†	175 (64.8)	95 (35.2)		199 (73.7)	71 (26.3)	
Shop/Market	34 (73.9)	12 (26.1)		36 (78.3)	10 (21.7)	
Bar/Lodge/Hotel	42 (60.0)	28 (40.0)		50 (71.4)	20 (28.6)	
Farming	52 (71.2)	21 (28.8)		56 (76.7)	17 (23.3)	
Housewife	40 (60.6)	26 (39.4)		52 (78.8)	14 (21.2)	
Others††	82 (60.3)	54 (39.7)	0.378	104 (76.5)	32 (23.5)	0.882
Marital status						
Not married	129 (61.7)	80 (38.3)		144 (68.9)	65 (31.1)	
Married monogamous	208 (64.0)	117 (36.0)		250 (76.9)	75 (23.1)	
Married polygamous	88 (69.3)	39 (30.7)	0.369	103 (81.1)	24 (18.9)	0.026
Education						
None	16 (47.1)	18 (52.9)		27 (79.4)	7	
Primary	245 (64.6)	134 (35.4)		286 (75.5)	93	
Secondary or beyond	164 (66.1)	84 (33.9)	0.091	184 (74.2)	64	0.790
Religion						
Catholic	163 (63.7)	93 (36.3)		198 (77.3)	58 (22.7)	
Protestant/Anglican	107 (62.9)	63 (37.1)		130 (76.5)	40 (23.5)	
Muslim	96 (65.8)	50 (34.2)		107 (73.3)	39 (26.7)	
Pentecostal/Adventist	59 (66.3)	30 (33.7)	0.926	62 (69.7)	27 (30.3)	0.470
Ethnicity/Tribe						
Baganda	217 (66.8)	108 (33.2)		254 (78.2)	71 (21.8)	
Others	208 (61.9)	128 (28.1)	0.192	243 (72.3)	93 (27.7)	0.083
Duration in community at enrolment (years)						
Less than 2	46 (54.1)	39 (45.9)		49 (57.7)	36 (42.3)	
2–4	159 (61.4)	100 (38.6)		182 (70.3)	77 (29.7)	
5–10	129 (67.2)	63 (32.8)		160 (83.3)	32 (16.7)	
More than 10	91 (72.8)	34 (27.2)	0.024	106 (84.8)	19 (15.2)	< 0.0001

†Fishing, fishmongers, fish processing, boat maker/owner

††Construction/Mechanic/Government/Clerical

Overall, 74.9% (495/661) participants attained good retention (Table 3), and it was significantly higher in the physical tracing arm compared to the phone arm (78.5% versus 71.2%, *p* = 0.03) and among island based participants relative to mainland dwellers (79.2% versus 71.1%, *p* = 0.016). After adjusting for sex, age, education, occupation, community location, length of stay in the community, and marital status in a logistic regression analysis, the odds of good retention were 1.56 (95% CI;1.08–2.26, *p* = 0.018) for physical contact tracing compared to mobile phone tracing (Table 3). The adjusted odds of good retention were statistically higher among island dwellers than mainland participants, AOR = 1.62 (95% CI; 1.08–2.43, *p* = 0.019), and in participants who had stayed in the fishing communities for 5–10 years, AOR = 2.88 (95% CI; 1.58–5.28, *p* = 0.001) and for more than 10 years, AOR = 3.46 (95% CI; 1.78–6.73, *p* < 0.0001), relative to those with less than one year of stay. Statistically borderline higher adjusted differences in good retention were observed for females compared to males, AOR = 1.70 (95% CI; 0.99–2.91, *p* = 0.052) and for participants aged 20–24 years relative to those aged 15–19 years, AOR = 1.99 (95% CI; 0.98–4.04, *p* = 0.057).

**Table 3:** Proportions and odds ratios of good retention by study arm and socio-demographic characteristics

	All follow up visits			
	Good retention (5/6 of 6 visits) n (%)		Odd Ratios (95% CI)	
	Yes	No	Unadjusted	Adjusted
All participants	495 (74.9)	166 (25.1)	–	–
Study arm				
Phone tracing	235 (71.2)	95 (28.8)	1 (reference)	1
Physical tracing	260 (78.5)	71 (21.4)^¶^	1.48 (1.04–2.11)^¶^	1.56 (1.08–2.26)^¶^
Sex				
Male	295 (73.2)	108 (26.8)	1	1
Female	200 (77.5)	58 (22.5)	1.26 (0.87–1.82)	1.70 (0.99–2.91)^ϕ^
Age (years)				
15–19	31 (63.3)	18 (36.7)	1	1
20–24	134 (76.6)	41 (23.4)	1.90 (0.96–3.74)	1.99 (0.98–4.04)^ϕ^
25–34	238 (74.4)	82 (25.6)	1.68 (0.89–3.17)	1.54 (0.78–3.04)
35–49	92 (78.6)	25 (21.4)	2.14 (1.03–4.43)^¶^	1.67 (0.76–3.67)
Education				
None	25 (73.5)	9 (26.5)	1	1
Primary	284 (74.9)	95 (25.1)	1.08 (0.48–2.39)	1.19 (0.52–2.75)
Secondary and beyond	173 (75.2)	57 (24.8)	1.08 (0.48–2.44)	1.31 (0.55–3.10)
Occupation				
Fishing/Fishing related^a^	204 (75.6)	66 (24.4)	1	1
Shop/Market	35 (76.1)	11 (23.9)	1.03 (0.49–2.14)	0.75 (0.32–1.75)
Bar/Lodge/Hotel	49 (70.0)	21 (30.0)	0.75 (0.42–1.35)	0.60 (0.28–1.27)
Farming	61 (83.6)	21 (16.4)	1.64 (0.83–3.24)	1.29 (0.63–2.66)
Housewife	50 (75.8)	16 (14.2)	1.01 (0.54–1.89)	0.76 (0.33–1.72)
Others^b^	96 (70.6)	40 (29.4)	0.78 (0.49–1.23)	0.76 (0.47–1.24)
Community location				
Mainland	251 (71.1)	102 (28.9)	1	1
Island	244 (79.2)	64 (20.8)^¶^	1.55 (1.08–2.22)^¶^	1.62 (1.08–2.43)^¶^
Duration of stay (years)				
Less than 1	51 (60.0)	34 (40.0)	1	1
– 4	188 (72.6)	71 (27.4)	1.76 (1.06–2.95)^¶^	1.70 (0.99–2.92)^ϕ^
– 10	155 (80.7)	37 (19.3)	2.79 (1.59–4.90)^¶^	2.88 (1.58–5.28)^¶^
More than 10	101 (80.8)	24 (19.2)	2.80 (1.51–5.22)^¶^	3.46 (1.78–6.73)^¶^
Marital statu**s**				
Not married	152 (72.7)	57 (27.3)	1	1
Married monogamous	246 (75.7)	79 (24.3)	1.17 (0.78–1.73)	0.93 (0.59–1.44)
Married polygamous	97 (76.4)	30 (23.6)	1.21 (0.73–2.02)	0.88 (0.49–1.55)

^¶^Statistically significant at *p* < 0.05, ^ϕ^Borderline statistical significance 0.06<*p* > 0.05, ^a^Fishing, fishmongers, fish processing, boat maker/owner, ^b^Construction/Mechanic/Government/Clerical

## Discussion

In this individual randomized trial among residents of fishing communities on Lake Victoria, Uganda, we found that participants’ reminders via physical contact tracing yielded statistically significant higher retention rates than mobile phones. This observation occurred with regard to the proportion of scheduled visits completed overall, and after the first six months of follow-up when retention was assessed by month of study visit, adjusting for adjusting for sex, age, education, occupation, community location, length of stay in the community and marital status. Good retention (defined as completion of 5 out of 6 scheduled follow-up visits) was significant higher in the physical contact reminder arm, among island dwellers and participants who had stayed in the fishing communities for 5 or more years.

We think that physical contact tracing performed better than mobile phone because the following reasons. While physical contact reminders are time and energy consuming and require effort on the part of the tracer, at individual level this approach provides direct contact with participants which in turn allows for dialogue on visit attendance and any other study related issues. Those advantages seem to work well particularly in the latter part of follow-up when excitement about continued participation in the study may have waned due to fatigue and or study-related unwanted effects. At interpersonal level, physical tracing provides room for other persons (family, workmates, community members) to inform the participant that a mobilizer was looking for him/her in case the former was absent at the time of tracing. This is may inadvertently remind a participant about the visit since he/she could guess why the mobilizer was looking for him/her. In addition, in the small and closely knit communities in Africa, it is likely that a community member may alert a mobilizer about the presence of a person he/she was looking for earlier on and this would enable the mobilizer to make another attempt to remind a participant. These additional efforts are unlikely to occur in the phone arm given the privacy associated with phones. Furthermore, phone loss and unavailability due to lack of power at the time of reminders may have led to fewer reminders in the phone arm. Mobile phone technology (text messages or audio call) removes the need for physical contact, allows getting in touch with participants at a wide range of time (day or night) regardless of their physical location. Nonetheless, it has several disadvantages especially in developing countries. These include but are not limited to unreliable connectivity, lack of constant power to charge phones, temporary and permanent change of phone ownership, loss of phones, and inability to comprehend a message especially text messages [16, 17]. Other barriers to retention like lack of transport, inflexible work schedules and forgetting research appointments are addressed by both physical contact tracing and mobile phone reminders, so they may not explain the differences retention levels between the two approaches.

The overall scheduled visit completion rate in this study was similar to that observed in Thai RV 144 trial but higher than that observed among a female sex workers (FSWs) study in Barcelona that assessed retention in the absence of an actual vaccine trial [18]. The retention observed in the mobile phone arm of this study at 6 and 12 months in this study was higher than that reported in the phone arm among FSWs in Barcelona (85% versus 76% at month 6 and 82% versus 69% at month 12, respectively) and that found in the enhanced arm (SMS and phone call reminders or in-person reminders without a phone, 59%) in a randomized trial of repeat HIV testing an persons with acute HIV infection in Kenya [13, 15]. The differences could be partly explained by dissimilarities in study populations between this study and the one in Spain, and age group with the Kenyan trial which enrolled participants aged 18–29 years.

In this study we also found that only 64% of participants completed all the four follow-up visits in the early phase that corresponds with the period where vaccinations are administered. This was much lower than the 85% observed among women in the Thai RV 144 trial [19].

The strength of this study was the ability to test two modes enhancing participant retention through a randomized trial design, using a visit schedule that mimicked an HIV vaccine trial, in a HIV high-risk population that it potentially eligible for HIV vaccine efficacy trials. However, there were some limitations. First, there was blinding of the intervention and we did not track cross-over of intervention. Although physical contact tracers were strictly warned and repeatedly reminded against using phones to track participants, there was a possibility of that happening and if it occurred it could have diluted the difference between the study arms. Second, there were no actual investigational products administered so the assessment of retention, even though it followed a visit schedule typically seen in actual HIV vaccine trials, was hypothetical. Third, we did not conduct formal qualitative assessments on participants’ perspectives on being contacted using either of the two different methods or both in a hybrid process where those not traceable via mobile phones would have physical contact tracing and vice versa. We also did not formally assess the community health mobilizers’ experiences on physical contact tracing. However, informally many mobilizers told us that participants always thanked them about the reminders and indicated they had forgotten about the visits. Such qualitative data would have enhanced the interpretation of our findings. Lastly, retention was assessed in absence of actual HIV vaccine trial and the hypothetical findings may not necessarily translate into actual retention in real HIV vaccine trial willingness observed in real vaccine trials [20, 21].

## Conclusion

Among fishing community populations of Lake Victoria, Uganda, 84% of follow-up visits can be attained and participant retention is higher using physical contact reminders than mobile phones. Regardless of method of reminding, persons residing on islands and those whose duration of stay in the fishing communities was five years or more have significantly higher follow-up rates.

## Abbreviations

ART: Anti-Retroviral Therapy
FSWs: Female Sex Workers
HIV: Human Immuno-deficiency Virus
LTFU: Loss to Follow-Up
PCR: Polymerase Chain Reaction
RNA: Ribonucleic Acid
SMS: Short message service
UNAIDS: The Joint United Nations Programme on HIV/AIDS
